# DELTA^2^ guidance on choosing the target difference and undertaking and reporting the sample size calculation for a randomised controlled trial

**DOI:** 10.1186/s13063-018-2884-0

**Published:** 2018-11-05

**Authors:** Jonathan A. Cook, Steven A. Julious, William Sones, Lisa V. Hampson, Catherine Hewitt, Jesse A. Berlin, Deborah Ashby, Richard Emsley, Dean A. Fergusson, Stephen J. Walters, Edward C. F. Wilson, Graeme Maclennan, Nigel Stallard, Joanne C. Rothwell, Martin Bland, Louise Brown, Craig R. Ramsay, Andrew Cook, David Armstrong, Doug Altman, Luke D. Vale

**Affiliations:** 1 Centre for Statistics in Medicine, Nuffield Department of Orthopaedics, Rheumatology and Musculoskeletal Sciences, University of Oxford, Botnar Research Centre, Nuffield Orthopaedic Centre, Windmill Rd, Oxford, OX3 7LD UK; 2 Medical Statistics Group, ScHARR, The University of Sheffield, Regent Court, 30 Regent Street, Sheffield, S1 4DA UK; 3 Statistical Methodology and Consulting, Novartis, Basel, Switzerland; 4 Department of Mathematics and Statistics, Lancaster University, Lancaster, LA1 4YF UK; 5 Department of Health Sciences, Seebohm Rowntree Building, University of York, Heslington, York, YO10 5DD UK; 6 Johnson & Johnson, 1125 Trenton-Harbourton Road, Titusville, NJ 08933 USA; 7 Imperial Clinical Trials Unit, School of Public Health, Imperial College London, Stadium House, 68 Wood Lane, London, W12 7RH UK; 8 Department of Biostatistics and Health Informatics, Institute of Psychiatry, Psychology and Neuroscience, King’s College London, De Crespigny Park, Denmark Hill, London, SE5 8AF UK; 9 Clinical Epidemiology Program, Ottawa Hospital Research Institute, Ottawa, ON Canada; 10 Cambridge Centre for Health Services Research & Cambridge Clinical Trials Unit, University of Cambridge, Institute of Public Health, Forvie Site, Robinson Way, Cambridge, CB2 0SR UK; 11 The Centre for Healthcare Randomised Trials (CHaRT), Health Sciences Building, University of Aberdeen, Foresterhill, Aberdeen, AB25 2D UK; 12 Warwick Medical School - Statistics and Epidemiology, University of Warwick, Coventry, CV4 7AL UK; 13 MRC Clinical Trials Unit at UCL, Institute of Clinical Trials & Methodology, 2nd Floor 90 High Holborn, London, WC1V 6LJ UK; 14 Health Services Research Unit, University of Aberdeen, Health Sciences Building Foresterhill, Aberdeen, AB25 2ZD UK; 15 Public Health Medicine and Fellow in Health Technology Assessment, Wessex Institute, University of Southampton, Alpha House, Enterprise Road, Southampton, SO16 7NS UK; 16 School of Population Health & Environmental Sciences, Faculty of Life Sciences and Medicine, Kings College London, Addison House, Guy’s Campus, London, SE1 1UL UK; 17 Health Economics Group, Institute of Health & Society, Newcastle University, Newcastle upon Tyne, NE2 4AX UK

## Abstract

**Background:**

A key step in the design of a RCT is the estimation of the number of participants needed in the study. The most common approach is to specify a target difference between the treatments for the primary outcome and then calculate the required sample size. The sample size is chosen to ensure that the trial will have a high probability (adequate statistical power) of detecting a target difference between the treatments should one exist.

The sample size has many implications for the conduct and interpretation of the study. Despite the critical role that the target difference has in the design of a RCT, the way in which it is determined has received little attention. In this article, we summarise the key considerations and messages from new guidance for researchers and funders on specifying the target difference, and undertaking and reporting a RCT sample size calculation. This article on choosing the target difference for a randomised controlled trial (RCT) and undertaking and reporting the sample size calculation has been dual published in the *BMJ* and *BMC Trials* journals

**Methods:**

The DELTA^2^ (Difference ELicitation in TriAls) project comprised five major components: systematic literature reviews of recent methodological developments (stage 1) and existing funder guidance (stage 2); a Delphi study (stage 3); a two-day consensus meeting bringing together researchers, funders and patient representatives (stage 4); and the preparation and dissemination of a guidance document (stage 5).

**Results and Discussion:**

The key messages from the DELTA^2^ guidance on determining the target difference and sample size calculation for a randomised caontrolled trial are presented. Recommendations for the subsequent reporting of the sample size calculation are also provided.

## Background

Properly conducted, the RCT is generally considered to be the gold standard for assessing the comparative clinical efficacy and effectiveness of healthcare interventions, as well as providing a key source of data for estimating cost-effectiveness [1]. These trials are routinely used to evaluate a wide range of treatments and have been successfully used in a variety of health and social care settings. Central to the design of a RCT is an *a-priori* sample size calculation, which ensures the study has a high probability of achieving its pre-specified objectives.

The difference between groups used to calculate a sample size for the trial, the “target difference”, is the magnitude of difference in the outcome of interest that the RCT is designed to reliably detect. Reassurance in this regard is typically confirmed by having a sample size which has a sufficiently high level of statistical power (typically 80 or 90%) for detecting a difference as big as the target difference, while setting the statistical significance at the level planned for the statistical analysis (usually this is the 2-sided 5% level). A comprehensive methodological review conducted by the original DELTA (Difference ELicitation in TriAls) group [2, 3] highlighted the available methods and limitations in current practice. It showed that despite there being many different approaches available, some are used only rarely in practice [4]. The initial DELTA guidance does not fully meet the needs of funders and researchers. The overall aim of the DELTA^2^ project, commissioned by the UK Medical Research Council (MRC)/National Institute for Health Research (NIHR) Methodology Research Programme (MRP), and described here, was to produce updated guidance for researchers and funders on specifying and reporting the target difference (“effect size”) in the sample size calculation of a RCT. In this article, we summarise the process of developing the new guidance, as well as the relevant considerations, key messages and recommendations for determining and reporting a RCT’s sample size calculation (Tables 1 and 2). This article on choosing the target difference for a randomised controlled trial (RCT) and undertaking and reporting the sample size calculatio has been dak published in tge BMJ and BMC Trials journals.

**Table 1:** DELTA^2^ recommendations undertaking a sample size calculation and choosing the target difference for a RCT

Begin by searching for relevant literature to inform the specification of the target difference. Relevant literature can:	
a. relate to a candidate primary outcome and/or the comparison of interest, and;	
b. inform what is an important and/or realistic difference for that outcome, comparison and population.	
2. Candidate primary outcomes should be considered in turn, and the corresponding sample size explored. Where multiple candidate outcomes are considered, the choice of the primary outcome and target difference should be based upon consideration of the views of relevant stakeholders groups (for example, patients), as well as the practicality of undertaking such a study with the required sample size. The choice should not be based solely on which yields the minimum sample size. Ideally, the final sample size will be sufficient for all key outcomes though this is not always practical.	
3. The importance of observing a particular magnitude of a difference in an outcome, with the exception of mortality and other serious adverse events, cannot be presumed to be self-evident. Therefore, the target difference for all other outcomes requires additional justification to infer importance to a stakeholder group.	
4. The target difference for a definitive (e.g. Phase III) trial should be one considered to be important to at least one key stakeholder group.	
5. The target difference does not necessarily have to be the minimum value that would be considered important if a larger difference is considered a realistic possibility or would be necessary to alter practice.	
6. Where additional research is needed to inform what would be an important difference, the anchor and opinion seeking methods are to be favoured. The distribution method should not be used. Specifying the target difference based solely upon a Standardised Effect Size approach should be considered a last resort though it may be helpful as a secondary approach.	
7. Where additional research is needed to inform what would be a realistic difference, the Opinion Seeking and the Review of the Evidence Base methods are recommended. Pilot trials are typically too small to inform what would be a realistic difference and primarily address other aspects of trial design and conduct.	
8. Use existing studies to inform the value of key “nuisance” parameters which are part of the sample size calculation. For example, a pilot trial can be used to inform the choice of the standard deviation value for a continuous outcome and the control group proportion for a binary outcome, along with other relevant inputs such as the amount of missing outcome data.	
9. Sensitivity analyses, which consider the impact of uncertainty around key inputs (e.g. the target difference and the control group proportion for a binary outcome) used in the sample size calculation, should be carried out.	
10. Specification of the sample size calculation, including the target difference, should be reported according to the guidance for reporting items (see below) when preparing key trial documents (grant applications, protocols and result manuscripts).

**Table 2:**
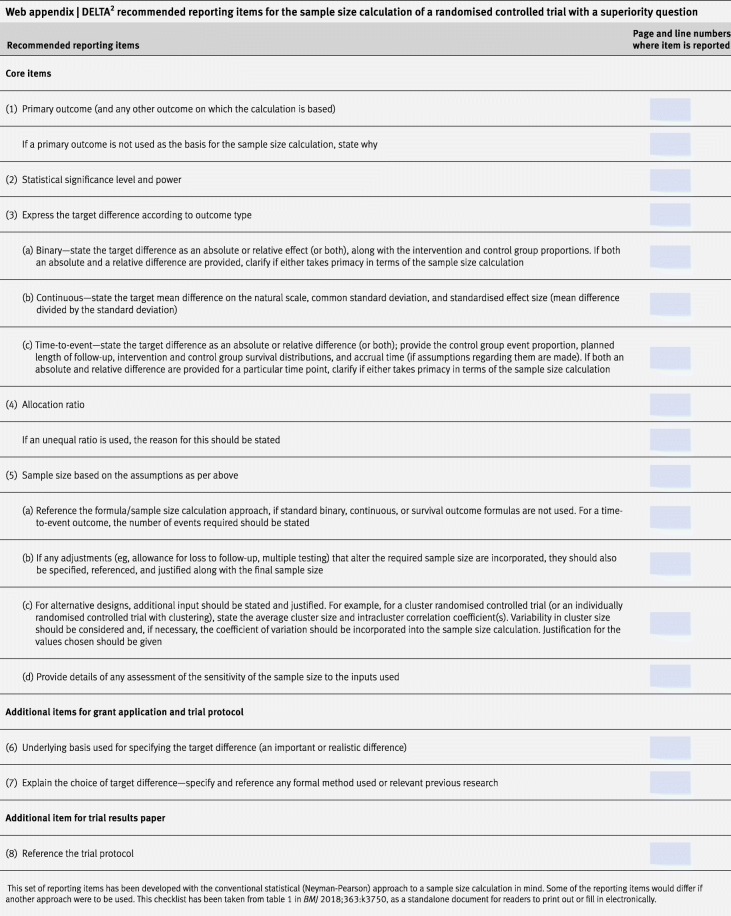
DELTA^2^ recommended reporting items for the sample size calculation of a RCT with a superiority question

### Development of the DELTA^2^ guidance

**Fig. 1:**
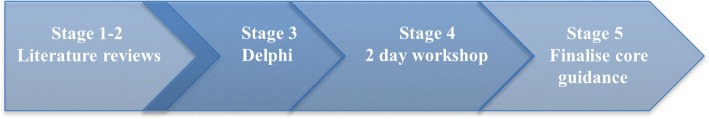
DELTA2 project components of work

The DELTA^2^ guidance is the culmination of a five stage process to meet the stated project objectives (see Fig. 1) which included two literature reviews of existing funder guidance and recent methodological literature, a Delphi process to engage with a wider group of stakeholders, a 2 day workshop and finalising the core guidance.

The literature review was conducted between April and December 2016 (searching up to April 2016). The Delphi study had two rounds: one held in 2016 before a two-day workshop in Oxford (September 2016) and another between August and November 2017. The general structure of the guidance was devised at the workshop. It was substantially revised based upon feedback from stakeholders received through the Delphi study. In addition, stakeholder engagement events were held at various meetings throughout the development of the guidance: the Society for Clinical Trials (SCT) meeting, and Statisticians in the Pharmaceutical Industry (PSI) conferences both in May 2017, Joint Statistical Meeting (JSM) in August 2017 and a Royal Statistical Society (RSS) Reading local group meeting in September 2017. These interactive sessions provided feedback on the scope (in 2016) and then draft guidance (in 2017). The core guidance was provisionally finalised in October 2017 and reviewed by the funders’ representatives for comment (MRP advisory group). The guidance was further revised and finalised in February 2018. The full guidance document incorporating case studies and relevant appendices is available here [5]. Further details on the findings of the Delphi study and the wider engagement with stakeholders are reported elsewhere [6]. The guidance and key messages are summarised in the remainder of the paper.

### The target difference and sample size calculations in RCTs

The role of the sample size calculation is to determine how many patients are required for the planned analysis of the primary outcome to be informative. It is typically achieved by specifying a target difference for the key (primary) outcome which can be reliably detected and the required sample size calculated. In this summary paper we restrict considerations to the most common trial design looking at a superiority question (one which assumes no difference and looks for a difference), although the full guidance considers equivalence and non-inferiority designs which invert the hypothesis and how the use of the target difference differs for such designs [5].

The precise research question that the trial is primarily set up to answer will determine what needs to be estimated in the planned primary analysis, this is known formally as the ‘estimand’. A key part of deciding this is choosing the primary outcome, which requires careful consideration. The target difference should be a difference that is appropriate for that estimand [7–10]. Typically (for superiority trials), an “intention to treat” or treatment policy estimand - that is, according to the randomised groups irrespective of subsequent compliance with the treatment allocation - is used. Other analyses that address different estimands [8, 9, 11] of interest (e.g. those based on the effect upon receipt of treatment and the absence of non-compliance) could also inform the choice of sample size. Different stakeholders can have somewhat differing perspectives on the appropriate target difference [12]. However, a key principle is that the target difference should be one that would be viewed as important by at least one (and preferably more) key stakeholder groups that is, patients, health professionals, regulatory agencies, and healthcare funders. In practice, the target difference is not always formally considered and in many cases appears, at least from trial reports, to be determined upon convenience, the research budget, or some other informal basis [13]. The target difference can be expressed as an absolute difference (e.g., mean difference or difference in proportions) or a relative difference (e.g., hazard or risk ratio), and it is also often referred to, rather imprecisely, as the trial “effect size”.

Statistical calculation of the sample size is far from an exact science [14]. Firstly, investigators typically make assumptions that are a simplification of the anticipated analysis. For example, the impact of adjusting for baseline factors is very difficult to quantify upfront, and even though the analysis is intended to be an adjusted one (such as when randomisation has been stratified or minimised), [15] the sample size calculation is often conducted based on an unadjusted analysis. Secondly, the calculated sample size can be sensitive to the assumptions made in the calculations such that a small change in one of the assumptions can lead to substantial change in the calculated sample size. Often a simple formula can be used to calculate the required sample size. The formula varies according to the type of outcome, how the target difference is expressed (e.g. a risk ratio versus a difference in proportions), and somewhat implicitly the design of the trial and the planned analysis. Typically, a sample size formula can be used to calculate the required number of observations in the analysis set, which varies depending on the outcome and the intended analysis. In some situations, ensuring the sample size is sufficient for more than one planned analysis may be appropriate.

When deciding upon the sample size for a RCT, it is necessary to balance the risk of incorrectly concluding there is a difference when no actual difference between the treatments exists, with the risk of failing to identify a meaningful treatment difference when the treatments do differ. Under the conventional approach, referred to as the statistical hypothesis testing framework [16], the probabilities of these two errors are controlled by setting the significance level (Type I error) and statistical power (1 minus Type II error) at appropriate levels (typical values are 2 sided 5% significance and 80% or 90% power respectively). Once these two inputs have been set, the sample size can be determined given the magnitude of the between group difference in the outcome it is desired to detect (the target difference). The calculation (reflecting the intended analysis) is conventionally done on the basis of testing for a difference of any magnitude. As a consequence, it is essential when interpreting the analysis of a trial to consider the uncertainty in the estimate, which is reflected in the confidence interval. A key question of interest is what magnitude of difference can be ruled out. The expected (predicted) width of the confidence interval can be determined for a given target difference and sample size calculation which is a helpful further aid in making an informed choice about this part of a trial’s design [17]. Other statistical and economic approaches to calculating the sample size have been proposed such as precision and Bayesian based approaches, [16, 18–20] and value of information analysis, [21] though they are not at present commonly applied [22].

The required sample size is very sensitive to the target difference. Under the conventional approach, halving the target difference quadruples the sample size for a two arm 1:1 parallel group superiority trial with a continuous outcome [23]. Appropriate sample size formulae vary depending upon the proposed trial design and statistical analysis, although the overall approach is consistent. In more complex scenarios, simulations may be used but the same general principles hold. It is prudent to undertake sensitivity calculations to assess the potential effect of misspecification of key assumptions (such as the control response rate for a binary outcome or the anticipated variance of a continuous outcome).

The sample size calculation and the target difference, if well specified, help provide reassurance that the trial is likely to detect a difference at least as large as the target difference in terms of comparing the primary outcome between treatments. Failure to clarify sufficiently what is important and realistic at the design stage can lead to subsequent sample size revisions, an unnecessarily inconclusive trial due to lack of statistical precision, or to ambiguous interpretation of the findings [24, 25]. When specifying the target difference with a definitive trial in mind, the following guidance should be considered.

### Specifying the target difference for a randomised controlled trial

Different statistical approaches can be taken to specify the target difference and calculate the sample size but the general principles are the same. To aid those new to the topic and to encourage better practice and reporting regarding the specification of the target difference for a RCT, a series of *recommendations* is provided in Tables 1 and 2. Seven broad types of methods can be used to justify the choice of a particular value as the target difference: these are summarised in Table 3.

**Table 3:** Methods that can be used to inform the choice of the target difference

*Methods that inform what is an important difference*	
*Anchor:* The outcome of interest can be “anchored” by using either a patient’s or health professional’s judgement to define what an important difference is. This may be achieved by comparing a patient’s health before and after treatment and then linking this change to participants who showed improvement/deterioration using a more familiar outcome (for which either patients or health professionals more readily agree on what amount of change constitutes an important difference).. Contrasts between patients (e.g., individuals with varying severity of a disease) can also be used to determine a meaningful difference.	
*Distribution:* Approaches that determine a value based upon distributional variation. A common approach is to use a value that is larger than the inherent imprecision in the measurement and therefore likely to represent a minimal level needed for a noticeable difference.	
*Health economic:* Approaches that use principles of economic evaluation. These compare cost with health outcomes, and define a threshold value for the cost of a unit of health effect that a decision-maker is willing to pay, to estimate the overall incremental net benefit of one treatment versus the comparator. A study can be powered to exclude a zero incremental net benefit at a desired statistical significance and power. A radically different approach is a (Bayesian) decision-theoretic value of information analysis which compares the added value with the added cost of the marginal observation, thus avoiding the need to specify a target difference.	
*Standardised effect size:* The magnitude of the effect on a standardised scale defines the value of the difference. For a continuous outcome, the standardised difference (most commonly expressed as Cohen’s d “effect size”, the mean difference dividing by the standard deviation) can be used. Cohen’s cutoffs of 0.2, 0.5, and 0.8 for small, medium, and large effects, respectively, are often used. Thus a “medium” effect corresponds simply to a change in the outcome of 0.5 SDs. When measuring a binary or survival (time-to-event) outcome alternative metrics (e.g., an odds, risk, or hazard ratio) can be utilised in a similar manner, though no widely recognised cut-points exist. Cohen’s cut-points approximate odds ratios of 1.44, 2.48, and 4.27, respectively. Corresponding risk ratio values vary according to the control group event proportion.	
*Methods that inform what is a realistic difference*	
*Pilot study:* A pilot (or preliminary) study may be carried out where there is little evidence, or even experience, to guide expectations and determine an appropriate target difference for the trial. In a similar manner, a Phase 2 study could be used to inform a Phase 3 study though this would need to take account of methodological differences (e.g. inclusion criteria and outcomes) that should be reflected in specification of the target difference.	
*Methods that inform what is an important and/or a realistic difference*	
*Opinion-seeking:* The target difference can be based on opinions elicited from health professionals, patients, or others. Possible approaches include forming a panel of experts, surveying the membership of a professional or patient body, or interviewing individuals. This elicitation process can be explicitly framed within a trial context.	
*Review of evidence base:* The target difference can be derived from current evidence on the research question. Ideally, this would be from a systematic review or meta-analysis of RCTs. In the absence of randomised evidence, evidence from observational studies could be used in a similar manner.	

Broadly speaking, two different approaches can be taken to specify the target difference for a RCT. A difference that is considered to be:

- *important* to one or more stakeholder groups
- *realistic* (plausible), based on either existing evidence, or expert opinion.

A very large literature exists on defining and justifying a (clinically) important difference, particularly for quality of life outcomes [26–28]. In a similar manner, discussions of the relevance of estimates from existing studies are also common; there are a number of potential pitfalls to their use, which requires careful consideration of how they should inform the choice of the target difference [2]. It has been argued that a target difference should always be both important and realistic [29], which would seem particularly apt when designing a definitive (Phase III) superiority RCT. In a sample size calculation for a RCT, the target difference between the treatment groups, strictly relates to a group level difference for the anticipated study population. However, the difference in an outcome that is important to an individual might differ from the corresponding value at the population level. More extensive consideration of the variations in approach is provided elsewhere [3, 30].

### Reporting the sample size calculation

The approach taken when determining the sample size and the assumptions made should be clearly specified. This information should include all the inputs and formula or simulation results, so that it is clear what the sample size was based upon. This information is critical for reporting transparency, allows the sample size calculation to be replicated, and clarifies the primary (statistical) aim of the study. Under the conventional approach with a standard (1:1 allocation two arm parallel group superiority) trial design and unadjusted statistical analysis, the core items needed to be stated are the primary outcome, the target difference appropriately specified according to the outcome type, the associated “nuisance” parameter (that is, a parameter that, together with the target difference, uniquely specifies the difference on the original outcome scale—eg, the event rate in the control group for a binary primary outcome), and the statistical significance and power. More complicated designs can have additional inputs that also need considered, like the intra-cluster correlation for a cluster randomised design.

A set of core items should be reported in all key trial documents (grant applications, protocols and main results papers) to ensure reproducibility and plausibility of the sample size calculation. The full list of recommended core items are given in Table 2 which is an update of the previously-proposed list [31]. When the sample size calculation deviates from the conventional approach, whether by research question or statistical framework, the core reporting set may be modified to provide sufficient detail to ensure the sample size calculation is reproducible and the rationale for choosing the target difference is transparent. However, the key principles remain the same. If the sample size is determined based upon a series of simulations, this would need to be described in sufficient detail to enable equivalent level of transparency and assessment. Additional items to give more explanation of the rationale should be provided where space allows (e.g. grant applications and trial protocols). Trial result publications can then reference these documents if sufficient space is not available to provide a full description.

## Discussion

Researchers are faced with a number of difficult decisions when designing a RCT, the most important of which are the choice of trial design, primary outcome and sample size. The latter is largely driven by the choice of the target difference, although other aspects of sample size determination also contribute.

The DELTA^2^ guidance provides help on specifying a target difference and undertaking and reporting the sample size calculation for a RCT. The guidance was developed in response to a growing recognition from funders, researchers, as well as other key stakeholders (such as patients and the respective clinical communities) of a real need for practical and accessible advice to inform a difficult decision. The new guidance document therefore aims to bridge the gap between the existing (limited) guidance and this growing need.

The key message for researchers is the need to be more explicit about the rationale and justification of the target difference when undertaking and reporting a sample size calculation. Increasing focus is being placed upon the target difference in the clinical interpretation of the trial result, whether statistically significant or not. Therefore the specification and reporting of the target difference, and other aspects of the sample size calculatio, needs to be improved.

## Abbreviations

BMC: BioMed Central
BMJ: British Medical Journal
DELTA: Difference ELicitation in TriAls
JSM: Joint Statistical Meeting
MRC: Medical Research Council
MRP: Methodology Research Programme
NIHR: National Institute for Health Research
PSI: Statisticians in Pharmaceutical Industry
RCT: Randomised Controlled Trial
RSS: Royal Statistical Society
SCT: Society for Clinical Trials
